# Work-limiting musculoskeletal pain and its association with loss of paid employment among senior workers: prospective cohort study with register follow-up

**DOI:** 10.1093/eurpub/ckad090

**Published:** 2023-06-09

**Authors:** Sebastian Venge Skovlund, Jonas Vinstrup, Emil Sundstrup, Lars Louis Andersen

**Affiliations:** Department of Musculoskeletal Disorders and Physical Workload, National Research Centre for the Working Environment, Copenhagen, Denmark; Department of Sports Science and Clinical Biomechanics, University of Southern Denmark, Odense, Denmark; Department of Musculoskeletal Disorders and Physical Workload, National Research Centre for the Working Environment, Copenhagen, Denmark; Department of Musculoskeletal Disorders and Physical Workload, National Research Centre for the Working Environment, Copenhagen, Denmark; Department of Musculoskeletal Disorders and Physical Workload, National Research Centre for the Working Environment, Copenhagen, Denmark; Department of Health Science and Technology, Aalborg University, Aalborg, Denmark

## Abstract

**Background:**

A growing population of elderly necessitates a sharpened focus on sustainable employment through aging. Physically demanding work can be challenging, especially for senior workers. Establishing determinants of labor market participation could guide policy development and preventive efforts at the workplaces aiming at keeping senior workers longer in the labor market.

**Methods:**

We used data from SeniorWorkingLife, a comprehensive questionnaire survey among a representative sample of Danish +50-year workers, and investigated the prospective association between self-reported work limitations due to musculoskeletal pain (‘work-limiting pain’) in 2018 and register-based loss of paid employment before state pension age at 2-year follow-up among +50-year Danish workers with physically demanding work (*n* = 3050).

**Results:**

Results showed that work-limiting pain increased the risk of loss of paid employment before the state pension age in a progressive manner, i.e. the higher degree of work-limiting pain, the higher risk of loss of paid employment (*P* < 0.001). Experiencing a low degree of work-limiting pain was associated with an 18% increased risk of loss of paid employment [risk ratio (RR): 1.18, 95% confidence interval (CI): 1.14–1.21], whereas experiencing a very high degree of work-limiting pain increased the risk of loss of paid employment by 155% (RR: 2.55, 95% CI: 2.43–2.69) compared to no work-limiting pain.

**Conclusion:**

In conclusion, work-limiting pain constitutes an important risk factor for loss of paid employment among senior workers with physically demanding work, and effective preventive efforts at both policy and workplace levels should be documented and implemented.

## Introduction

In light of the projected increase in the population of elderly people, several industrialized countries have increased the statutory retirement age in parallel with reforms to motivate people—through economic means—to work longer. This may not be without challenges, as some people, especially those with physically demanding work, may find it challenging to work until a high age.^1^ Establishing determinants of labor market participation could guide policy development and preventive efforts at the workplaces aiming at increasing labor market participation among senior workers with physically demanding work.

Multiple factors are known to influence labor market participation, and reduced labor market participation is often a complex and dynamic process that can be more or less voluntary and have different origins.^2–7^ At macro level, societal structures such as economic (dis)incentives can have profound impact on labor market participation.^4^^,^^6^ Likewise, ergonomic and psychosocial working conditions have also been reported as important determinants.^2^^,^^6^ Furthermore, individual circumstances related to health, private life and leisure have also shown to influence labor market participation. For instance, factors such as more time for hobbies and freedom to choose what to do during leisure have been reported as prominent expected reasons for retiring.^3^ Contrastingly, poor health and the feeling of inability to adequately perform work tasks have been reported as prominent expected reasons for early retirement, especially among workers with physically demanding work.^3^ In accordance, a systematic review identified poor health as an important risk factor for premature exit from paid employment through different routes, i.e. disability pension, unemployment and early retirement.^7^

Having a musculoskeletal disorder (MSD) is a well-established risk factor for reduced labor market participation through, e.g. disability pension.^7–12^ Notably, workers with physically demanding work are at substantially greater risk of developing MSDs^13^^,^^14^ and reduced labor market participation^15^ compared to workers with more sedentary jobs. Additionally, both the incidence and prevalence of health problems such as MSDs increase with age,^16^ with prevalences of daily musculoskeletal pain as high as 40–50% among senior workers with physically demanding work.^14^ Interestingly, a recent study showed that the negative health consequences of physically demanding work increase with age,^17^ and it is hence particularly crucial to focus on sustainable employment among senior workers with physically demanding work.

Previous studies have investigated the impact of MSDs on labor market participation.^7–12^^,^^18^ However, as the manifestation of MSDs vary highly between individuals, the resulting practical consequences may differ accordingly, including the degree to which the MSD affects activities of daily living and labor market participation. In fact, multiple factors have been shown to influence the practical consequences of MSDs, e.g. musculoskeletal pain intensity^8^^,^^19^ and physical work demands.^20^ This underscores the importance and potential value of investigating the risk of experiencing practical consequences of MSDs on future labor market participation. To the best of our knowledge, this has barely been investigated to date. Wilkie and coworkers reported an increased risk of premature work loss among workers experiencing osteoarthritis pain-mediated work interference.^21^ Therefore, the current analyses seek to guide preventive strategies aiming at reducing risk factors for reduced labor market participation, and, thereby, facilitate sustainable employment among senior workers with physically demanding work.

Drawing on data from SeniorWorkingLife (SWL), we investigated the prospective association between self-reported work limitations due to musculoskeletal pain (‘work-limiting pain’) in 2018 and register-based loss of paid employment before state pension age among +50-year workers with physically demanding work.

## Methods

### Study design and participants

This prospective cohort study with follow-up in a high-quality national register is part of the SWL project. SWL has been registered in ClinicalTrials.gov (ID number: NCT03634410), and the methodology has been described thoroughly previously.^1^^,^^22^^,^^23^ In brief, SWL is a questionnaire-based cohort study about work environment and health among Danish senior workers. A probability sample of 30 000 Danes at and above 50 years was drawn by Statistics Denmark (the central authority on Danish statistics) and received the baseline questionnaire electronically between July and October 2018.^23^ In this study, only currently employed wage earners with physically demanding work were included.

Of the 30 000, 18 000 currently employed wage earners received the baseline questionnaire,^23^ whereof 56% completed the entire questionnaire (*n* = 9974). However, not all participants filled in all survey questions, and therefore the exact number of participants for each analysis differ. As reported previously,^23^ we defined ‘currently employed wage earners’ based on the following criteria: (i) paid employment for at least 20 hours per week during March 2018 *and* for at least half of the months during the preceding year as of March 2018, and (ii) not having received economic benefits for, e.g. sickness absence or maternity/paternity leave during the first quarter of 2018.^23^

Data on the predictor variable was available for 11 786 workers. Physical work demands were assessed by the following question: ‘How would you describe the physical activity level in your current job?’, with the following four response options: (i) ‘Mostly sedentary work that is not physically demanding’, (ii) ‘Mostly standing and walking work that otherwise is not physically demanding’, (iii) ‘Standing or walking work with some lifting and carrying tasks’ and (iv) ‘Heavy or fast work that is physically demanding’.^1^^,^^2^^,^^6^ For the present analyses, we included workers responding 3 or 4 (*n* = 3050). Of these, replies on the predictor variable was available for 3030 workers. Reporting of the study followed the guidelines for the reporting of observational studies in epidemiology (STROBE).^24^

### Explanatory variables

#### Work-limiting pain

To assess work-limiting musculoskeletal pain, participants replied to the following question: ‘How often have you experienced musculoskeletal pain during the past 3 months?’, with these response options given: (i) ‘Every day’, (ii) ‘One or several times a week’, (iii) ‘A couple of times a month’, (iv) ‘A couple of times’ (v) ‘Not at all’. If participants replied having musculoskeletal pain ‘A couple of times’ or more frequently during the last three months, i.e. response options 1–4, they received the question about work-limiting musculoskeletal pain, a modified version of the Standardized Nordic Questionnaire for Musculoskeletal Symptoms^25^:

‘To which degree did the pain limit you in your work during the previous 3 months?’. The response options were (i) ‘To a very high degree’, (ii) ‘To a high degree’, (iii) ‘To some degree’, (iv) ‘To a low degree’ and (5) ‘Not at all’. If participants replied ‘Not at all’ on the first question about pain frequency, these were coded to having not having work-limiting pain (‘Not at all’).

### Outcome variable

#### Loss of paid employment before state pension age

The register-based outcome measure, loss of paid employment, has been described previously^26^^,^^27^ and was defined as workers no longer fulfilling the abovementioned criteria for being employed during the 2-year follow-up period from 2018 to 2020 (see ‘Study design and participants’). We truncated at the baseline age of 63 to ensure that the participants had loss of paid employment before the official state pension age, which in Denmark was 65 years in 2018 (raised to 66 in 2020). Hence, the outcome describes loss of paid employment *before state pension age* and it encompasses individuals with loss of paid employment either permanently or temporarily for voluntary or involuntary reasons, or those who substantially reduced their working hours before state pension age, e.g. through unemployment or disability pension.

### Control variables

Analyses were controlled for the following control variables that have previously been associated with loss of paid employment through disability pension, unemployment and early retirement from the labor market^7^^,^^10^^,^^28–30^: age (years), gender (male/female), educational level (unskilled, skilled, further education, see below), body mass index (BMI: kg/m^2^), smoking status (Yes/No), musculoskeletal pain (‘Yes, daily or weekly’/’No, not at all or a couple of times a month’) and psychosocial work factors using two items adapted from the validated Copenhagen Psychosocial Questionnaire on influence at work and recognition from colleagues.^31^ Highest attained educational level was drawn from a national register by Statistics Denmark: (i) Primary school or high school (unskilled), (ii) Vocational education (skilled), (iii3) Longer-term further education.

### Ethical approval

In accordance with Danish law, questionnaire- and register data can be used for scientific purposes without collecting informed consent or approval by ethical and scientific committees. Statistics Denmark depersonalized and stored all data on their servers, and researchers performed analyses through remote access.

### Statistical analyses

We used the GLIMMIX procedure to produce risk ratios (RR) for the association between the predictor variable work-limiting pain, and the outcome loss of paid employment before state pension age, controlling for various confounders (Proc Glimmix, SAS version 9.4, SAS Institute, Cary, NC). To account for different sizes and response percentages of subgroups and thus make estimates representative, we used model-assisted statistical weights based on information from high-quality national registers (provided by Statistics Denmark) and accounted for age, gender, highest completed education, occupational industry, family type, family income and origin. Applying the weight variable in the GLIMMIX procedure repairs both non-response and deviations of the probability sample from the general working population, and we therefore did not impute data.^23^ We applied two statistical models. Statistical model 1 was adjusted for age, gender and education, whereas model 2 was adjusted for the same as model 1 as well as for BMI, smoking status, musculoskeletal pain and psychosocial work factors. We also performed a subgroup analysis to test whether the risk of loss of paid employment associated with work-limiting pain differed across genders (interaction analysis). Due to a statistically significant interaction, these results were therefore reported stratified by gender. Results are presented as RR and 95% confidence intervals (CIs) unless otherwise stated, with alpha levels below 0.05 considered statistically significant.

## Results

Table 1 reports baseline characteristics of the sample. Of the included 3050 senior workers with physically demanding work, the mean age was 56 years whereof 46% were women. Approximately a third of the workers did not experience work-limiting pain (31.3%, *n* = 955), whereas the remainder 68.7% had a low to a very high degree of work-limiting pain (*n* = 2075). During the follow-up period from 2018 to 2020, 15.7% of the study sample (*n* = 734) lost paid employment.

**Table 1:** Descriptive baseline characteristics of the study sample

	*n*	%	Mean	SD
Age (years)	3050		56.0	4.5
Gender	3050			
Men	1766	54.3		
Women	1284	45.7		
Education	3050			
Unskilled	801	27.6		
Skilled	1756	55.8		
Further education	493	16.6		
Smoking	3042			
No	2265	75.6		
Yes	777	24.4		
BMI (kg/m^2^)	3021			
<18	26	0.9		
18−<25	1136	38.4		
25−<30	1253	41.1		
30−<35	459	14.5		
35−<40	114	3.7		
≥40	33	1.3		
Musculoskeletal pain	3033			
Yes, daily or weekly	1850	60.4		
No, not at all or a couple of times a month	1183	39.6		
Collegial recognition (0–100)	3045			
0–25	125	4.2		
25–75	499	16.1		
75–100	2421	79.7		
Influence at work (0–100)	3050			
0–25	162	5.2		
25–75	875	28.6		
75–100	2013	66.2		
Work-limiting pain	3030			
To a very high degree	90	2.8		
To a high degree	173	6.1		
To some degree	719	22.7		
To a low degree	1093	37.2		
Not at all	955	31.3		

*Note*: *n*, number; %, percentage; SD, standard deviation.

Table 2 presents the prospective association between work-limiting pain and loss of paid employment before state pension age. In both statistical models, higher degrees of work-limiting pain were associated with loss of paid employment in a progressive manner (*P* < 0.001). After full adjustment, even a low degree of work-limiting pain was associated with an 18% increased risk of loss of paid employment (RR: 1.18, 95% CI: 1.14–1.21), whereas experiencing a very high degree of work-limiting pain increased the risk of loss of paid employment by 155% (RR: 2.55, 95% CI: 2.43–2.69) compared to no work-limiting pain. A significant interaction existed between gender and work-limiting pain for the risk of loss of paid employment. Among both women and men, higher degrees of work-limiting pain generally increased the risk of loss of paid employment. However, the risk was generally higher among men compared to women. Among men, the risk was significantly increased at all degrees of work-limiting pain, whereas women reporting a ‘low degree’ of work-limiting pain were not at significantly elevated risk of loss of paid employment (RR: 0.94, 95% CI: 0.90–0.98). Still, women experiencing a ‘very high’ degree of work-limiting pain demonstrated the highest risk of loss of paid employment (RR: 3.23, 95% CI: 3.05–3.43), also compared to men with similar (very high) degree of work-limiting pain (RR: 2.01, 95% CI: 1.84–2.21).

**Table 2:** Associations between work-limiting pain and loss of paid employment

Loss of paid employment					
		Model 1	**Model 2** **(all)**	**Model 2** **(women)**	Model 2(men)
Work-limiting pain	Loss of paid employment *n* (%)	RR (95% CI)	RR (95% CI)	RR (95% CI)	RR (95% CI)
Not at all (reference)	197 (0.9 %)	1	1	1	1
To a low degree	241 (1.6%)	1.23 (1.20–1.27)	1.18 (1.14–1.21)	0.94 (0.90–0.98)	1.48 (1.41–1.55)
To some degree	199 (4.0%)	1.44 (1.40–1.49)	1.38 (1.33–1.43)	1.23 (1.17–1.28)	1.62 (1.53–1.71)
To a high degree	58 (5.4%)	2.22 (2.13–2.31)	2.14 (2.04–2.23)	1.60 (1.49–1.71)	2.81 (2.64–2.99)
To a very high degree	39 (3.8%)	2.81 (2.69–2.94)	2.55 (2.43–2.69)	3.23 (3.05–3.43)	2.01 (1.84–2.21)

*Note*: *n* (%) = absolute number of the sample and weighted percentage of the population of senior workers with physically demanding work with loss of paid employment during follow-up. RR = risk ratio. 95% CI = 95% confidence Intervals. Model 1 was adjusted for age, gender and education, whereas model 2 was adjusted for the same as model 1 as well as for BMI, smoking status, musculoskeletal pain and psychosocial work factors. Model 2 stratified by gender was adjusted for all the same variables as model two including both women and men, except for gender.

## Discussion

This study showed that the degree of work-limiting pain is progressively associated with higher risk of loss of paid employment among senior workers with physically demanding work. Preventing or reducing pain and, more importantly, the practical consequences of pain in form of work limitations, seem imperative to secure sustainable employment in the years to come among this particularly vulnerable group of senior workers.

### Comparison to previous studies

In line with previous studies on the relationship between MSD and labor market participation,^7^^,^^8^^,^^11^^,^^12^^,^^18^^,^^21^ our results confirm that indications of poor health—here work-limiting pain—constitute important risk factors for reduced labor market participation among both women and men. In contrast to our study, previous studies have differentiated between exit routes, and reported that MSDs primarily increase the risk of reduced labor market participation through disability pension,^7^^,^^8^^,^^11^^,^^12^ whereas the evidence is more conflicting as pertains to unemployment and early retirement.^7^^,^^10–12^

Our study assessed the relationship between work-limiting pain—a potential work-related/practical consequence of MSD—and loss of paid employment among male and female workers. Similarly to our study, Wilkie and colleagues investigated determinants of premature work loss among senior workers (≥50 years) consulting primary healthcare for osteoarthritis (*n* = 612).^21^ Premature work loss was defined as retirement prior to state retirement age or transition from employment to being off work due to health or unemployment. After adjustment for age, gender, socio-economic position and work conditions, workers with moderate to extreme osteoarthritis-induced pain interference on their normal work were at 51% increased risk of premature work loss over the 6-year study period compared to workers without or with only minor osteoarthritis pain interference.^21^ Our results indicate an increased risk of loss of paid employment among men compared to women experiencing work-limiting pain. One possible explanation could be that the labor market in Denmark is still somewhat gender-segregated, e.g. more men work in construction work and more women in social and care work like eldercare. Some typically male-dominated industries like construction work may be more susceptible to economic fluctuations and job losses due to downsizing. This complicates a direct comparison of results between men and women, and it further justifies the gender-stratified analysis. In comparison, Wilkie and colleagues reported male gender as a significant risk factor for premature work loss, but did not assess the interaction between gender and pain interference for the risk of premature work loss.^21^ Other studies have found no gender differences in the risk of exit from paid employment among workers with MSD.^11^^,^^18^

Our study focused specifically on senior workers with physically demanding work, as this group is at elevated risk of MSDs^13^^,^^14^ and reduced labor market participation^15^ compared to workers with more sedentary jobs. Furthermore, the negative health consequences of physically demanding work have been shown to increase with age.^17^ Thus, in light of the expected demographic changes, these findings emphasize the necessity of increased focus on sustainable employment among especially senior workers with physically demanding work.

### Implications

The reported progressive association between work-limiting pain and loss of paid employment indicates that even small improvements in work-limiting pain could be of great significance for the individual workers. Biopsychosocial and multidisciplinary approaches may have the potential to reduce work-limiting pain and retain labor market participation among the large number of particularly senior workers with work limitations due to MSDs.^32^ As both high pain intensity and low physical capacity have been associated with increased risk of work-limiting pain^20^^,^^33^ and reduced labor market participation,^8^^,^^34^ improving any of these by physical exercise performed either at the workplace or during leisure could pose a viable prevention strategy.^35^ Another viable strategy could be to reduce the demands from work and thereby increase work ability by improving the balance between individual capacity and work demands. Here physical and psychosocial work conditions may play a role. Physically demanding work has been linked with increased risks of work limitations due to pain^20^ and reduced labor market participation among workers with MSDs,^36^ but contradicting null-findings have also been reported.^21^ Thus, tailoring the physical work demands to the capacity of the worker with a MSD, e.g. through increased use of technical assistive devices, increasing rest, or re-organization of work tasks, could be a solution in some cases, and these elements could be included in senior policies at the workplaces. It is reasonable to assume that the largest effect could come from improving both the individual worker’s capacity *and* reducing the work demands. An increased focus on—both politically and at the workplaces—and possibility for lifelong learning could also allow for transition to a less physically demanding occupation, which has been associated with reduced risk of disability pension,^37^ likely due to better matching of work capacity and demands. A good psychosocial work environment could also lower the risk of reduced labor market participation, as poor psychosocial work conditions, including low control and high psychological demands, have been associated with an increased risk of disability pension.^38^ In addition, good coworker support has been associated with a lower risk of premature work loss among workers with osteoarthritis.^21^ Improving individual resources such as self-efficacy may also protect against the negative consequences of MSDs. Mine and coworkers found that high pain intensity was associated with impaired work function in a dose–response fashion among construction employees, but that high pain self-efficacy was protective for the ill consequences of high pain intensity, i.e. workers with high self-efficacy was not in elevated risk of impaired work function, even among workers with severe pain.^33^

Notably, the present study design does not allow to conclude on the effect of specific interventions on work-limiting pain and labor market participation. Future intervention studies should investigate the effect of preventive strategies aiming at increasing physical capacity in parallel with reducing work demands among senior workers with work-limiting pain on future labor market participation. Based on the existing literature, both national policies aimed at prolonging working lives as well as factors related to the work environment and organization may be viable strategies to improve labor market participation among senior workers with work-limiting pain and physically demanding work. Unfortunately, a strong inequality exists in opportunities offered by many workplaces for supporting a long and healthy work life, where (senior) workers with physically demanding work—those needing it the most—are generally offered fewer opportunities compared to sedentary workers.^22^ This poses a threat to prolonging working lives among senior workers with physically demanding work.

### Study strengths and limitations

Our study contains several strengths. First, the nationally representative sample of senior workers with physically demanding work—due to model-assisted statistical weighting—increases the generalizability and practical relevance as this group has the greatest challenges in terms of both MSDs, sickness absence, and reduced labor market participation. Another strength is the prospective design with register-based follow-up, and not self-reported labor market participation or *expected* reasons for losing paid employment, which can be susceptible to bias. Hence, the use of high-quality national registers reduces the risk of e.g. information bias.

Our study also contains several limitations. Inevitably, observational study designs do not allow causal inferences. Similar to the perception of pain, our predictor variable ‘work-limiting pain’ is a subjective experience influenced by a complex interaction of a multitude of factors—both biological, psychosocial and social—that also takes into account the practical consequence of pain on the performance of everyday work tasks. Our predictor variable thus relied on self-reported data, which can be influenced by e.g. the respondent’s mood and health status, inaccurate and biased, including recall bias.^39^ Furthermore, our data do not allow differentiating different routes of loss of paid employment, e.g. disability pension, unemployment or reduced working hours, for which risk factors could differ. However, while losing paid employment before the state pension age is likely more reflective of involuntary push mechanisms, few workers may have chosen this voluntarily. Unmeasured and residual confounding also cannot be ruled out and may have influenced the associations, e.g. co-morbidity and discrimination. As we only included senior workers with physically demanding work, our findings cannot be extrapolated to the general working population. Our findings may also be biased by ‘the healthy worker effect’ as we only included currently employed senior workers and not those that already had left the labor market prematurely or changed to a less physically demanding job due to health problems such as MSDs. In this context, our estimates may be considered more conservative.

## Conclusion

Work limitations due to pain constitute a significant risk factor for reduced labor market participation among senior workers with physically demanding work. Preventing or reducing pain and, more importantly, the practical consequences of pain in form of work limitations, seem imperative to secure sustainable employment in the years to come among this particularly vulnerable group of workers.

## Data Availability

The authors encourage collaboration and use of the data by other researchers. Data are stored on the server of Statistics Denmark, and researchers interested in using the data for scientific purposes should contact the project leader Prof. Lars L. Andersen, lla@nfa.dk.
